# Similar Hemodynamic Signal Patterns Between Compact NIRS and 52-Channel NIRS During a Verbal Fluency Task

**DOI:** 10.3389/fpsyt.2021.772339

**Published:** 2021-12-16

**Authors:** Jinichi Hirano, Akihiro Takamiya, Yasuharu Yamamoto, Fusaka Minami, Masaru Mimura, Bun Yamagata

**Affiliations:** Department of Neuropsychiatry, Keio University School of Medicine, Tokyo, Japan

**Keywords:** near-infrared spectroscopy, compact NIRS, neuroimaging, neurofeedback, children

## Abstract

Multichannel near-infrared spectroscopy (NIRS), including 52-channel NIRS (52ch-NIRS), has been used increasingly to capture hemodynamic changes in the brain because of its safety, low cost, portability, and high temporal resolution. However, optode caps might cause pain and motion artifacts if worn for extended periods of time because of the weight of the cables and the pressure of the optodes on the scalp. Recently, a small NIRS apparatus called compact NIRS (cNIRS) has been developed, and uses only a few flexible sensors. Because this device is expected to be more suitable than 52ch-NIRS in the clinical practice for patients with children or psychiatric conditions, we tested whether the two systems were clinically comparable. Specifically, we evaluated the correlation between patterns of hemodynamic changes generated by 52ch-NIRS and cNIRS in the frontopolar region. We scanned 14 healthy adults with 52ch-NIRS and cNIRS, and measured activation patterns of oxygenated-hemoglobin [oxy-Hb] and deoxygenated-hemoglobin [deoxy-Hb] in the frontal pole while they performed a verbal fluency task. We performed detailed temporal domain comparisons of time-course patterns between the two NIRS-based signals. We found that 52ch-NIRS and cNIRS showed significant correlations in [oxy-Hb] and [deoxy-Hb] time-course changes in numerous channels. Our findings indicate that cNIRS and 52ch-NIRS capture similar task-dependent hemodynamic changes due to metabolic demand, which supports the validity of cNIRS measurement techniques. Therefore, this small device has a strong potential for clinical application with infants and children, as well as for use in the rehabilitation or treatment of patients with psychiatric disorders using biofeedback.

## Introduction

Near-infrared spectroscopy (NIRS) has been widely used as an appealing technology in the field of neuropsychiatry (1). NIRS has the following advantages: (1) completely non-invasive measurement, which facilitates repeated measurements; (2) higher temporal resolution (0.1 s resolution) than functional magnetic resonance imaging (fMRI); and (3) portability and compactness of the apparatus, enabling measurements under natural conditions with participants sitting in a comfortable chair. On the downside, the spatial resolution of NIRS is less than that of fMRI, which has homogeneous resolutions of 3 mm. Recently, multichannel NIRS, such as 52 channel NIRS (52ch-NIRS), has greatly improved anatomic specificity and has been widely used in research (1). However, over time, the weight of the cables and the pressure of the optodes on the scalp can cause pain and motion artifacts. An additional limitation of NIRS is the lack of access to deep cortical regions that are not on the surface of the brain. For example, NIRS cannot evaluate hemodynamic changes in brain regions located in the ventral and medial frontal cortices, or the basal ganglia, because too much light is absorbed at those distances from the scalp, making measurements impossible.

Based on these clinical advantages and disadvantages, multichannel NIRS is thought to be most useful for research with infants and children, and for rehabilitation or biofeedback treatment of psychiatric disorders (2). Recently, a small, portable NIRS apparatus called compact NIRS (cNIRS) has been developed. These devices have only a few flexible sensors to fit the curvature of the head, which are appropriate for monitoring brain functions in neonates or children, as well as for use in the rehabilitation or treatment of patients with psychiatric disorders using biofeedback (3). Additionally, when focusing on measuring hemodynamic changes in the frontal pole, cNIRS is more appropriate for prolonged recordings because it has fewer cables, which makes it lighter. The current study examined the potential usability of cNIRS. Specifically, we evaluated how well the observed patterns of hemodynamic changes in the frontopolar region correlated between multichannel NIRS and cNIRS while participants performed a cognitive task.

## Methods

### Participants

Fourteen healthy adults participated in this study (mean age: 35.9 ± 7.8, male/female: 5/9). Participants who had psychiatric conditions, neurological disorders, head injury, or alcohol or other substance dependence, were taking psychotropic medication were excluded from the study. Following an explanation of the study, we obtained written informed consent from all participants. This study was conducted with the approval of the ethics committee of Keio University Hospital.

### Verbal Fluency Task

The task used in the present study was similar to that in Takizawa et al. (4). Participants sat in a comfortable chair and were instructed to relax and keep still. The oxygenated-hemoglobin [oxy-Hb] and deoxygenated-hemoglobin [deoxy-Hb] changes were measured during a verbal fluency task (VFT; letter version), which comprised three blocks, such as a 30-s pre-task baseline, 60-s VFT, and a 60-s post-task baseline. For the pre- and post-task baseline periods, the participants were instructed to consecutively repeat the five Japanese vowels (/a/, /i/, /u/, /e/, and /o/) aloud.

During the task period, participants were instructed to produce as many nouns as possible, without using repetitions or proper nouns, beginning with a designated syllable. One of these three sets of initial syllables (A; /to/, /se/, /o/, B; /a/, /ki/, /ha/, C; /na/, /i/, /ta/) were presented in counterbalanced order to the participants. Initial syllables were changed every 20-s during the 60-s task. The linear subtraction method (task measurements minus pre- and post-task baselines) minimized the vocalization effects during the VFT. The total number of correct words generated during the VFT was taken as a measure of task performance. The order of measurements by 52ch-NIRS and cNIRS was randomized for all participants.

### 52-Channel NIRS Measurements

We used a 52ch-NIRS machine (ETG-4000 Optical Topography System; Hitachi Medical Co., Japan) with two wavelengths (695 and 830 nm) and a time resolution of 0.1 s to measure the relative changes in absorbed near-infrared light. These changes were transformed into changes of [oxy-Hb], [deoxy-Hb], and total-hemoglobin [total-Hb; oxy-Hb + deoxy-Hb] concentrations as indicators for brain activity using a modified Beer–Lambert law (5). The unit was mM × mm (i.e., the changes in the chromophore concentration depend on the path length of the near-infrared light). 52ch-NIRS includes 33 optodes, consisting of 17 light emitters and 16 detectors with an inter-optode distance of 30 mm.

### Compact NIRS Measurements

**Figure 1:**
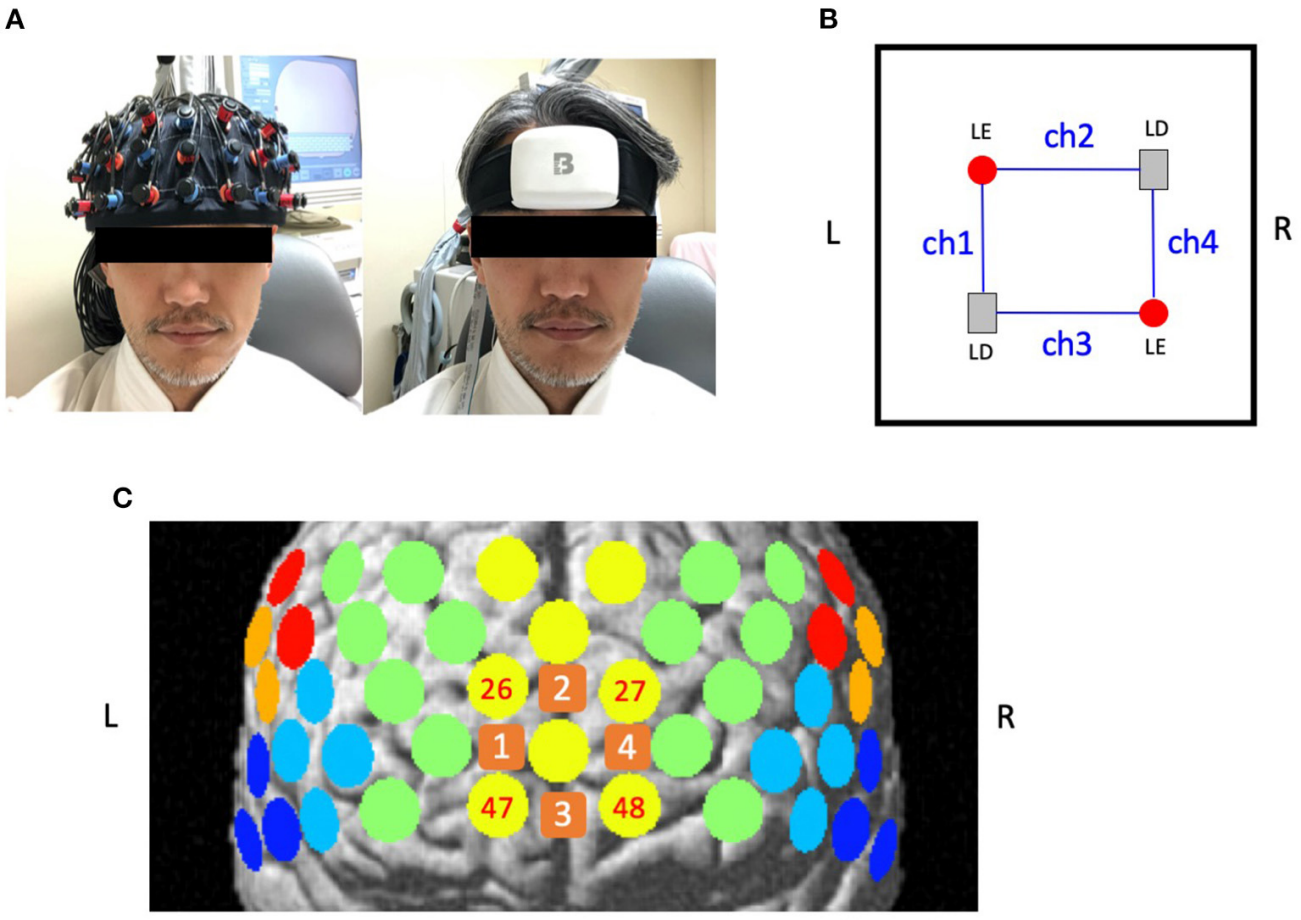
Location of channels for 52-channel near-infrared spectroscopy (52ch-NIRS) and compact NIRS (cNIRS). **(A)** The probe settings for 52ch-NIRS (left) and cNIRS (right). 52ch-NIRS covers broad regions from bilateral prefrontal to temporal cortices. cNIRS covers only the frontal pole region. **(B)** Location of 4 channels with 4 optodes (2 light emitters and 2 light detectors) in cNIRS. **(C)** Measuring positions for 52ch-NIRS and cNIRS are superimposed on the brain surface. Yellow circles represent 52ch-NIRS channels. Orange squares represent cNIRS channels. We created four cNIRS/52ch-NIRS channel pairs (ch1/ch26, ch2/ch27, ch3/ch47, and ch4/ch48). L, left; R, right; LE, light emitter; LD, light detector.

cNIRS (BrainEnergy System: BrainEnergy Inc. Tokyo, Japan) uses three different wavelengths (660, 800, and 940 nm) with a time resolution of 0.13 s. The device is 62 mm long, 82 mm wide, 28 mm thick, and weights 111 g (Figures 1A,B). It has only 4 channels with 4 optodes (2 light emitters and 2 detectors) with an inter-optode distance of 30 mm. cNIRS employs the same procedures as 52ch-NIRS to measure and calculate [oxy-Hb] and [deoxy-Hb].

### Preprocessing and Resampling Procedures

All data were analyzed using the “integral mode.” The pre-task baseline was determined as the mean over a 10-s period just prior to the task initiation, and the post-task baseline was determined as the mean over the last 5-s of the post-task period. Linear fitting was applied to the data between these two baselines. After applying linear fitting, to directly compare the data that was recorded at different temporal resolutions, the 52ch-NIRS data (sampling rate = 0.1 s) was linearly up-sampled to a sampling rate of 0.01 s and then down-sampled to 0.13 s, which was the sampling rate of the cNIRS data. Finally, these data were used in following correlation analyses.

### 52ch-NIRS and cNIRS Correations

A measuring point of activation (channel) was defined as the region between one emitter and one detector. Thus, the probe set for 52ch-NIRS covered brain regions that included the bilateral prefrontal {approximately dorsolateral [Brodmann's area (BA) 9, 46], ventrolateral [BA 44, 45, 47], and frontopolar [BA 10]}, superior temporal, and middle temporal cortical regions. The cNIRS optodes were set on the forehead located above the frontal pole (BA10). The correspondence between the optode positions and the measurement areas on the cerebral cortex was confirmed based on a previous multi-subject study of anatomical craniocerebral correction via the international 10-20 system (6, 7). Both 52ch-NIRS and cNIRS measured [oxy-Hb] and [deoxy-Hb] activation patterns during the VFT in the channels located in the bilateral frontal pole. In particular, we created the following four channel pairs between cNIRS and 52ch-NIRS (ch1/ch26, ch2/ch27, ch3/ch47, and ch4/ch48; Figure 1C) to compare the similarity of prefrontal activation patterns in the temporal domain. The probes were fastened to the head tightly by elastic straps.

For each of the four channel pairs for each participant, we calculated the Spearman correlation coefficients in the NIRS-based signal time-courses (for [oxy-Hb] and [deoxy-Hb]) between 52ch-NIRS and cNIRS. Overall, we obtained 56 correlation coefficients for both the [oxy-Hb] and [deoxy-Hb] measurements (4 channel pairs × 14 participants). Considering the statistical issue of multiple comparisons, we set the threshold for statistical significance at 0.00089 (0.05/56 pairs). Preprocessing (linear correction and resampling) and statistical analysis were conducted using in-house code based on the SciPy (https://www.scipy.org) package for python.

### Signal to Noise Ratio (SNR)

We calculated the signal to noise ratio (SNR) using the same methods of the previous NIRS study (8). In particular, the signal amplitude was defined as mean Hb changes during the VFT activation period and the noise amplitude was defined as the standard deviation of the Hb changes in pre-task baseline period. We obtained absolute SNR values by dividing signal by noise level and then compared these values between 52ch-NIRS and cNIRS in each channel pair using paired *t*-test. We set the threshold for statistical significance at 0.0125 (0.05/4 pairs).

## Results

**Figure 2:**
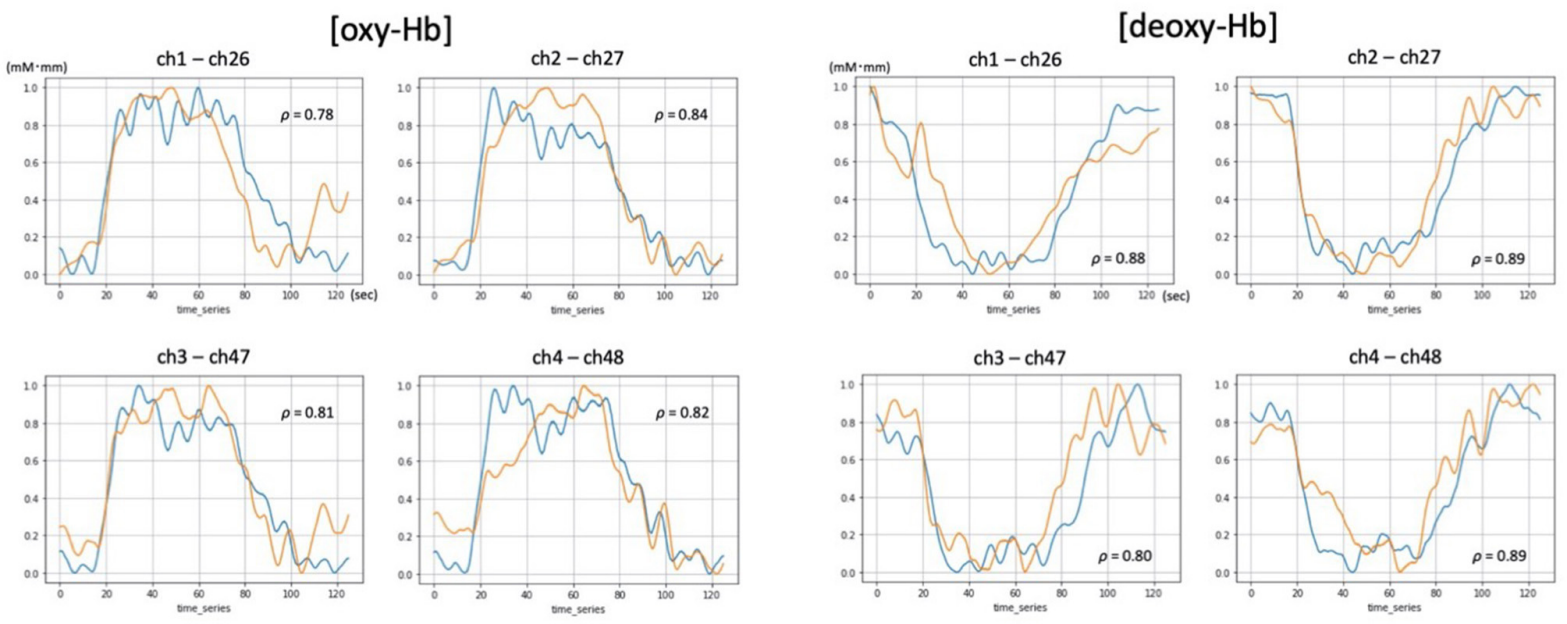
Examples of [oxy-Hb] and [deoxy-Hb] time-courses demonstrating significant and high correlations between 52ch-NIRS and cNIRS (*P*-value < 0.00089; correction for multiple comparisons). Blue and orange lines represent time course changes during the verbal fluency task (VFT) detected by 52ch-NIRS and cNIRS, respectively. For each of the four cNIRS and 52ch-NIRS channel pairs (ch1/ch26, ch2/ch27, ch3/ch47, and ch4/ch48), [oxy-Hb] and [deoxy-Hb] exhibited similar activation patterns. Transformed Hb-values (mM mm) ranged from 0 to 1 and are displayed for visualization purposes (the data were transformed using a minimum-maximum scaler, which converted the minimum value to 0 and the maximum value to 1 for each dataset).

**Figure 3:**
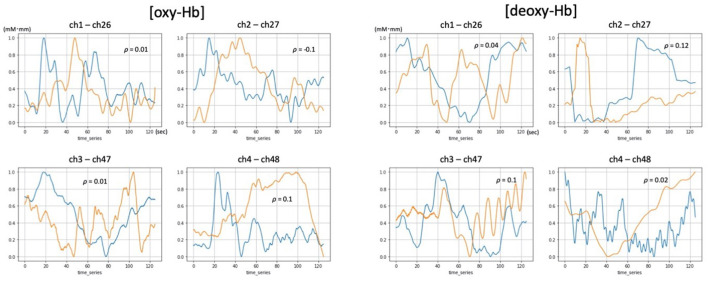
Examples of [oxy-Hb] and [deoxy-Hb] time courses demonstrating low correlation between 52ch-NIRS and cNIRS. Blue and orange lines represent time course changes during the verbal fluency task (VFT) detected by 52ch-NIRS and cNIRS, respectively. For each of the four cNIRS and 52ch-NIRS channel pairs (ch1/ch26, ch2/ch27, ch3/ch47, and ch4/ch48), each member of the pair exhibited quite distinct [oxy-Hb] and [deoxy-Hb] activation patterns. Transformed Hb-values (mM mm) ranged from 0 to 1 and are displayed for visualization purposes (the data were transformed using a minimum-maximum scaler, which converted the minimum value to 0 and the maximum value to 1 for each dataset).

We found that 52ch-NIRS and cNIRS correlations exhibited wide variability. For instance, at some channels in some people, correlation coefficients of NIRS parameters were as high as 0.8, while at other channels in other people, it was as low as 0.1 (Figures 2, 3). However, most channel pairs demonstrated significant correlations between 52ch-NIRS and cNIRS (*P* < 0.00089; corrected for multiple comparisons). In particular, 91% of channels (51/56) revealed significant time-course correlations in [oxy-Hb] and 77% of channels (43/56) showed significant time-course correlations in [deoxy-Hb]. Moreover, the means of the correlation coefficients were 0.44([oxy-Hb]) and 0.39([deoxy-Hb]) across all 56 pairs. We then confirmed that there were no significant differences in mean correlation coefficients between [oxy-Hb] and [deoxy-Hb] (*P* = 0.287). Furthermore, there were no significant differences of SNR between two devices in four channel pairs for both [oxy-Hb] and [deoxy-Hb] (Table 1).

**Table 1:** Comparisons of signal to noise ratio (SNR) between cNIRS and 52ch-NIRS.

**CH pair**	**Oxy-Hb**			**Deoxy-Hb**		
	**cNIRS**	**52ch-NIRS**	***P-*value**	**cNIRS**	**52ch-NIRS**	***P-*value**
ch1/ch26	10.85 ± 8.02	8.57 ± 7.71	0.503	8.87 ± 15.60	9.13 ± 9.61	0.956
ch2/ch27	13.06 ± 9.76	11.38 ± 21.61	0.757	10.35 ± 10.60	21.81 ± 54.05	0.408
ch3/ch47	7.19 ± 12.03	8.17 ± 10.49	0.837	5.42 ± 4.78	10.20 ± 12.45	0.210
ch4/ch48	19.53 ± 22.04	7.78 ± 9.55	0.116	6.95 ± 5.47	9.87 ± 12.8	0.414

*The table shows means ± standard deviations. Statistics show the results of comparisons between cNIRS and 52ch-NIRS. CH, channel; Oxy-Hb, oxygenated-hemoglobin; Deoxy-Hb, deoxygenated-hemoglobin; cNIRS, compact near-infrared spectroscopy; 52ch-NIRS, 52 channel near-infrared spectroscopy*.

## Discussion

The current study investigated how similar the patterns of hemodynamic change in the bilateral frontal pole were between 52ch-NIRS and cNIRS. We found that numerous channel pairs showed significant time-course correlations between these two devices in both [oxy-Hb] and [deoxy-Hb]. Our findings indicate that cNIRS captures task-dependent hemodynamic changes due to metabolic demand that are similar to those obtained via 52ch-NIRS, which supports the validity of cNIRS measurement techniques. Therefore, considering that it is smaller and more portable than the 52ch-NIRS, cNIRS has the potential for clinical application when focusing on forehead measurements.

Notably, significant time-course correlations were observed between two devices in the majority of channel pairs (91% of pairs for [oxy-Hb] and 77% of pairs for [deoxy-Hb]). The high correlation values might have resulted from using similar probe measurement systems and the same experimental paradigm. In addition, no significant SNR differences between two devices may also support our findings of high correlation values. In particular, distance from the scalp (and brain) does not differ significantly between the two systems among the optode pairs. Channel location for the devices was not exactly the same because of their structural differences, but because of the relatively low spatial resolution of NIRS (between 2 and 3 cm), we believe that the effect of location difference on [oxy-Hb] and [deoxy-Hb] activation patterns was minimal. A previous NIRS-fMRI correlation study demonstrated that longer distance from the scalp and brain negatively affects NIRS SNR and reduces NIRS ([oxy-Hb])-fMRI(BOLD) time-course correlations (9). Thus, channel locations for the frontal pole might have contributed to the high correlation values. In addition, frontal regions measured by NIRS probes on the forehead have the advantage of being hairless, which allows increased contact of the probes with the scalp and reduces artifacts. Furthermore, differences in the emitter-to-detector distance among probes are important because they can affect the depth of the photon path. We note that in our study, both devices measure hemodynamic changes using the same inter-optode distance of 30 mm.

Despite the overall results, we did find a wide range in correlation values. In particular, lower correlation values ranging from −0.1 to 0.1 were observed in 5 channel pairs for [oxy-Hb] and 10 channel pairs for [deoxy-Hb] (data not shown). As a potential reason of these observations, artifacts due to head motion might have contributed to these findings. Even though SNR is comparable between two devices, we assume that artifacts are more prevalent in 52ch-NIRS because its probe set covers broad regions from bilateral prefrontal to temporal cortices, which may induce lower correlation values. cNIRS has only 4 channels located in the frontal pole, is lighter and smaller than 52ch-NIRS, and fastening it tightly to the frontal scalp is easier. In multichannel NIRS, such as 52ch-NIRS, optode caps can become uncomfortable to wear over time because of the weight of the cables and the pressure of the optodes on the subject's scalp, which may induce more motion artifacts. Thus, reducing caps and cable weights by using cNIRS can help reduce these artifacts. Moreover, optimum processing for reducing artifacts of NIRS signals requires advanced knowledge of computer science, signaling processing, and engineering, which may make more accessible for use in the clinical environment.

Compared with other neuroimaging methods such as fMRI, NIRS has the disadvantage of lower spatial resolution and a lower SNR ratio (9). However, it has the advantage of higher temporal resolution because of its higher sampling rate. Furthermore, the practicality of NIRS is a major advantage over fMRI: it is easier to use, portable, safe, silent, inexpensive, and requires little setup time. Additionally, artifacts due to head motion occur less often in NIRS, which makes it possible to record brain activity in more natural situations. This is especially useful for patients with children or psychiatric conditions who are unable to keep still. Given these advantages of NIRS over other neuroimaging modalities, it has been increasingly used as a tool for neurofeedback (NF) training (10). During NF training, participants are trained to self-regulate their brain activity to achieve specific goals, with the aim of altering behavior or cognitive/emotional functions (11–13). Previous studies have indicated a clinical potential of NIRS-based NF training for psychiatric conditions such as attention deficit hyperactively disorder or autism spectrum disorder, however most of these studies used 52ch-NIRS (ETG-4000) (14). Because the brain regions targeted by NF in these studies were mainly distributed in part of the prefrontal cortex, cNIRS may be a more practical option for NF because of its smaller size and portability. This small device could allow the spread of NF treatment, especially if people can conduct the training at their homes with feedback via smartphone. Considering that we have not yet obtained a standard NF training protocol, cNIRS could make it possible to continue NF training at home for a long period, which might be effective for improving cognitive and emotional functions in patients.

This study had several limitations. First, fundamental system differences may affect our findings of lower correlation values in a few channel pairs. In particular, while both systems use the same theory of calculating [oxy-Hb] and [deoxy-Hb], they adopt distinct wavelengths, laser power, and sampling rates. Second, even though there were no significant differences in SNR among two devices, we observed relatively large variation in individual SNR data, which may be implicated in the low correlation values in some channels. Thus, considering statistical power due to relatively small sample size, the results of this exploratory analysis should be interpreted with caution and further research is warranted in a larger sample.

In summary, we revealed that 52ch-NIRS and cNIRS demonstrated significant and high correlations in the patterns of hemodynamic change detected in the bilateral frontal pole, suggesting that cNIRS and 52ch-NIRS are similarly able to capture task-dependent hemodynamic changes. These findings indicate that cNIRS has the potential to be a novel treatment method for psychiatric conditions.

## Data Availability Statement

The original contributions presented in the study are included in the article/supplementary material, further inquiries can be directed to the corresponding authors.

## Ethics Statement

The studies involving human participants were reviewed and approved by the Ethics Committee of Keio University Hospital. The patients/participants provided their written informed consent to participate in this study.

## Author Contributions

JH, MM, and BY designed the study and wrote the manuscript. Data were collected by JH, AT, YY, and FM. JH and BY analyzed and interpreted the data. All authors have approved the final manuscript.

## Funding

This work was supported by a project grant of BrainEnergy Inc.

## Conflict of Interest

MM has a potential conflict of interest; for the present study, BrainEnergy Inc. provided a project grant and material support [temporary rental of cNIRS (BrainEnergy System)]. The remaining authors declare that the research was conducted in the absence of any commercial or financial relationships that could be construed as a potential conflict of interest.

## Publisher's Note

All claims expressed in this article are solely those of the authors and do not necessarily represent those of their affiliated organizations, or those of the publisher, the editors and the reviewers. Any product that may be evaluated in this article, or claim that may be made by its manufacturer, is not guaranteed or endorsed by the publisher.
